# City-based action to reduce harmful alcohol use: review of reviews

**DOI:** 10.12688/f1000research.13783.2

**Published:** 2018-03-05

**Authors:** Peter Anderson, Eva Jané-Llopis, Omer Syed Muhammad Hasan, Jürgen Rehm

**Affiliations:** 1 Institute of Health & Society, Newcastle University, Baddiley-Clark Building, Richardson Road, Newcastle upon Tyne , NE2 4AX, UK; 2 Faculty of Health, Medicine and Life Sciences, Maastricht University, P. Debyeplein 1, Maastricht, 6221 HA , Netherlands; 3 Institute for Mental Health Policy Research, CAMH, 33 Russell Street, Toronto, ON M5S 2S1, Canada; 4 ESADE Business School, Ramon Llull University, Av. Esplugues 92-96, Barcelona, 08034, Spain; 5 Campbell Family Mental Health Research Institute, CAMH, 250 College Street, Toronto, ON M5T 1R8, Canada; 6 Institute of Medical Science (IMS) , University of Toronto, Medical Sciences Building,1 King’s College Circle, Room 2374, Toronto, ON M5S 1A8, Canada; 7 Department of Psychiatry, University of Toronto, 250 College Street, 8th Floor, Toronto, ON M5T 1R8, Canada; 8 Dalla Lana School of Public Health, University of Toronto, 155 College Street, 6th Floor, Toronto, ON M5T 3M7, Canada; 9 Institute for Clinical Psychology and Psychotherapy, TU Dresden, Chemnitzer Str. 46, Dresden, 01187 , Germany

**Keywords:** Cities; Municipalities; communities; harmful use of alcohol

## Abstract

**Background:** The World Health Organization global strategy on alcohol called for municipal policies to reduce the harmful use of alcohol. Yet, there is limited evidence that documents the impact of city-level alcohol policies.

**Methods: **Review of reviews for all years to July 2017. Searches on OVID Medline, Healthstar, Embase, PsycINFO, AMED, Social Work Abstracts, CAB Abstracts, Mental Measurements Yearbook, Health and Psychosocial Instruments, International Pharmaceutical Abstracts, International Political Science Abstracts, NASW Clinical Register, and Epub Ahead of Print databases. All reviews that address adults, without language or date restrictions resulting from combining the terms (“review” or “literature review” or “review literature” or “data pooling” or “comparative study” or “systematic review” or “meta-analysis” or “pooled analysis”), and “alcohol”, and “intervention” and (“municipal” or “city” or “community”).

**Results: **Five relevant reviews were identified. Studies in the reviews were all from high income countries and focussed on the acute consequences of drinking, usually with one target intervention, commonly bars, media, or drink-driving. No studies in the reviews reported the impact of comprehensive city-based action. One community cluster randomized controlled trial in Australia, published after the reviews, failed to find convincing evidence of an impact of community-based interventions in reducing adult harmful use of alcohol.

**Conclusions: **To date, with one exception, the impact of adult-oriented comprehensive community and municipal action to reduce the harmful use of alcohol has not been studied. The one exception failed to find a convincing effect. We conclude with recommendations for closing this evidence gap.

## Introduction

In response to the 2011 United Nations declaration on non-communicable diseases (NCDs) ( United Nations General Assembly, 2011; United Nations, 2014), the World Health Organization proposed a target to reduce the harmful use of alcohol by 10% between the years 2010 and 2025 ( World Health Organization, 2014a), based on three possible indicators, adult per capita alcohol consumption, prevalence of heavy episodic drinking, and measures of alcohol-related morbidity and mortality ( World Health Organization, 2014b).

The bulk of alcohol-related severe health problems, including mortality, occurs in middle age ( Office for National Statistics, 2015), and, it is amongst this age group that policy and programme interventions are likely to bring the greatest health and productivity gains ( Organisation for Economic Co-operation and Development, 2015). Heavy drinkers are responsible for the majority all alcohol-related harm ( Rehm *et al.*, 2013). It is also amongst this group, compared with lighter drinkers, that disproportionally greater health gains can be made for the same absolute reduction in alcohol consumption ( Rehm & Roerecke, 2013). Thus, if alcohol policy is to be most efficient in reducing the harmful use of alcohol, it should preferentially address adult drinkers, and, in particular, those who drink heavily.

Driving the NCD alcohol target are WHO’s Global Strategy to reduce the harmful use of alcohol ( World Health Organization, 2010), and WHO’s three ‘best buys’ ( World Economic Forum & World Health Organization, 2011; World Health Organization, 2013). One of the ten target areas within the global strategy is community action, with a specific call to: “strengthen capacity of local authorities to encourage and coordinate concerted community action by supporting and promoting the development of municipal policies to reduce harmful use of alcohol, as well as their capacity to enhance partnerships and networks of community institutions and nongovernmental Organizations” ( World Health Organization, 2010).

Cities are a potentially important setting and jurisdictional level for reducing NCDs ( De Leeuw *et al.*, 2015; Farrington *et al.*, 2015). There is a range of evidence-based interventions that fall within municipal jurisdictional responsibility and which could be implemented at city level to reduce the harmful use of alcohol ( Anderson & Baumberg, 2006; Anderson *et al.*, 2009; Anderson *et al.*, 2012; Burton *et al.*, 2017; Fitzgerald *et al.*, 2016; Martineau *et al.*, 2013). Despite a long history of calls for city-based policies and action plans ( Mathrani & Anderson, 1998; Ritson, 1995; World Health Organization, 1998), and an equally long history of research endeavour ( Giesbrecht *et al.*, 1990), a systematic review of the impact of alcohol policies, undertaken prior to the launch of the WHO global strategy, was unable to include community actions within its cost-effectiveness estimates, due to insufficient evidence of impact ( Anderson *et al.*, 2009).

Spurred by a target of a global beer producer to reduce the harmful use of alcohol by 10% over the five-year period 2016–2020 in pilot cities in at least nine different middle- and high-income countries ( ABInBev, 2016), we have undertaken a review of reviews to investigate the potential impact of city-based action to reduce the harmful use of alcohol amongst adults. In our review, we have focused on reviews that summarize the literature of comprehensive community and municipal action, often based on a municipal comprehensive strategy and action plan, as put forward by the World Health Organization ( Anderson, 1991; Ritson, 1995). Because we anticipated very few such reviews, we have supplemented our findings with an overview of what could be implemented within a comprehensive municipal strategy to reduce the harmful use of alcohol, based on the published evidence base ( Anderson & Baumberg, 2006; Anderson *et al.*, 2009; Anderson *et al.*, 2012; Burton *et al.*, 2017; Fitzgerald *et al.*, 2016; Martineau *et al.*, 2013).

## Methods

During July 2017, we conducted a systematic literature search on OVID Medline, Healthstar, Embase, PsycINFO, AMED, Social Work Abstracts, CAB Abstracts, Mental Measurements Yearbook, Health and Psychosocial Instruments, International Pharmaceutical Abstracts, International Political Science Abstracts, NASW Clinical Register, and Epub Ahead of Print databases to identify reviews that addressed community and municipal alcohol interventions. With no language or date restrictions, the search used the following combination of terms: (“review” or “literature review” or “review literature” or “data pooling” or “comparative study” or “systematic review” or “meta-analysis” or “pooled analysis”), and “alcohol” and “intervention” and (“municipal” or “city” or “community”), supplemented with hand searches of included reviews.

**Figure 1.**
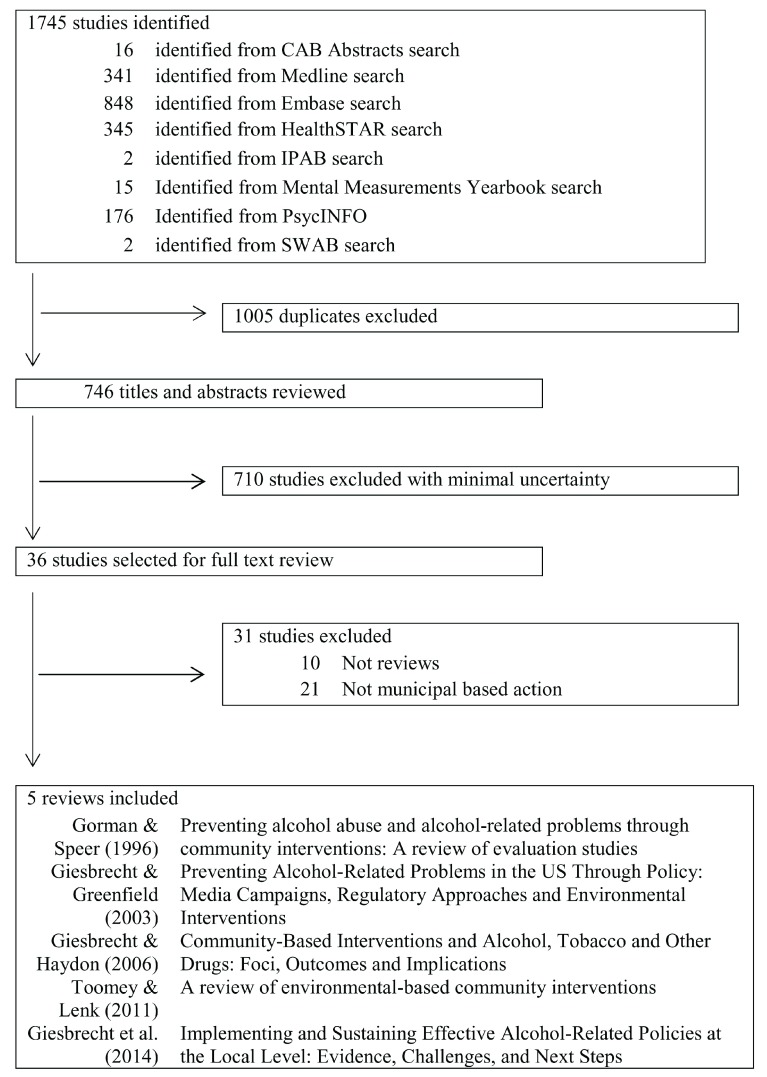
Flow chart for search of reviews.

Our inclusion criteria were reviews and overviews (whether or not systematic) that discussed the implementation of comprehensive policies and programmes to reduce the harmful use of alcohol amongst adults at the community or municipal level. We excluded reviews of specific alcohol policy interventions, for example restrictions on hours and days of sale, that may or may not have been part of a city action plan. Two authors (OSMH and PA) independently reviewed titles and abstracts for selecting papers for full text review and selecting papers to include. Discrepancies, which only related to whether or not the publication addressed comprehensive approaches, were resolved with discussion. The result of the search, analysed during July 2017, is summarized in Figure 1. As only five relevant reviews were identified, three of which were by the same author, we did not attempt to analyze them for their quality, but rather describe their methods and findings.

## Results

Only five relevant reviews were identified ( Giesbrecht *et al.*, 2014; Giesbrecht & Greenfield, 2003; Giesbrecht & Hayden, 2006; Gorman & Speer, 1996; Toomey & Lenk, 2001). Three included the same first author, and the most recent publication ( Giesbrecht *et al.*, 2014) included all publications of, and reached similar conclusions to, the previous four reviews. None of the reviews were systematic and none adhered to standard guidelines. Subsequent to the publication dates of the reviews, our search identified one further large randomized study of the effectiveness of community action ( Shakeshaft *et al.*, 2014).

Of the 23 individual studies mentioned in the 2014 review ( Giesbrecht *et al.*, 2014), twelve included adults, mostly with a focus on younger adults. Six were from North America, two were from Nordic countries, and four were from Australia/New Zealand. Four of the 12 studies targeted bars, three media campaigns, two drink-driving, one overall access, one a specific location (a beach), and one primary health care-based brief advice programmes. No studies reported comprehensive community or municipal interventions. The four bar studies found an effect in reducing violence, which tapered off over time. The three media studies found no impact. The two drink-driving studies led to reduced alcohol-involved traffic crashes. The access and location study led to reductions in the harmful use of alcohol, but the brief advice initiative did not.

The large study, outside of the reviews, was a randomized trial comprising 20 communities in Australia that each had populations of 5,000–20,000 inhabitants ( Shakeshaft *et al.*, 2014). Communities were pair-matched, and one member of each pair was randomly allocated to the experimental group. Thirteen interventions were implemented in the experimental communities from 2005 to 2009: community engagement; general practitioner training in alcohol screening and brief intervention (SBI); feedback to key stakeholders; media campaign; workplace policies/practices training; school-based intervention; general practitioner feedback on their prescribing of alcohol medications; community pharmacy-based SBI; web-based SBI; Aboriginal Community Controlled Health Services support for SBI; Good Sports program for sports clubs; identifying and targeting high-risk weekends; and hospital emergency department–based SBI. The study failed to detect an impact of the interventions in reducing the harmful use of alcohol in routine data, but found a significant reduction of average alcohol use in surveys. Unfortunately, the study appeared under powered for the routine data, and had low response rates to measurement surveys. The community interventions were not able to be fully comprehensive and were not able to include a range of potentially impactful interventions, including sales taxes and restrictions on availability. Further, no process evaluation was reported; thus, whether or not the included interventions were implemented as planned, or the extent to which they were implemented is not reported.

One of the five reviews that we identified included a summary of feasible intervention options at the local and municipal level ( Giesbrecht & Haydon, 2006). We have included and expanded on these in Table 1, adding an overview of the evidence base ( Anderson & Baumberg, 2006; Anderson *et al.*, 2009; Anderson *et al.*, 2012; Burton *et al.*, 2017; Fitzgerald *et al.*, 2016; Martineau *et al.*, 2013), with implementation illustrations at the city level. Dependent on jurisdictional responsibilities, city-level interventions that might have a meaningful contribution to reducing the harmful use of alcohol include sales taxes, restrictions on density of outlets and days and hours of sale, drink-drive restrictions, and scale-up of individual level advice and treatment programmes ( Organisation for Economic Co-operation and Development, 2015).

**Table 1.** Feasible adult-oriented policies and programmes implementable at city level dependent on jurisdictional responsibilities, with evidence of impact and opportunity for city implementation.

Policy measure	Evidence of impact	Opportunity for city implementation
Pricing policies		
Alcohol taxes	Effective Systematic reviews and meta-analyses find that increases in the price and taxation of alcohol reduce consumption and alcohol-related harm for all groups of drinkers, and in high, middle and low-income countries ( Anderson *et al.*, 2009; Dhalwani, 2011; Elder *et al.*, 2010; Fogarty, 2008; Gallet, 2007; Sornpaisarn *et al.*, 2013; Wagenaar *et al.*, 2009; Wagenaar *et al.*, 2010; Xu & Chaloupka, 2011).	Many cities have opportunity to set alcohol beverage sales taxes, which can bring in municipal revenues ( KPMG LLP, 2016).
Access policies		
Outlet density	Effective Systematic reviews ( Bryden *et al.*, 2012; Campbell *et al.*, 2009; Gmel *et al.*, 2016; Holmes *et al.*, 2014; Livingston *et al.*, 2007; Popova *et al.*, 2009) and individual studies ( Fone *et al.*, 2016; Morrison *et al.*, 2016; Richardson *et al.*, 2015) find that greater alcohol outlet density is associated with increased alcohol consumption and harms, including injuries, violence and crime.	Licensing of alcohol sales outlets allows local governments to control where alcohol is sold to the public, with restrictions on density related to less crime ( de Vocht *et al.*, 2016).
Days and hours of sale	Effective Systematic reviews find that days and hours of sale are related to alcohol consumption and harms ( Hahn *et al.*, 2010; Middleton *et al.*, 2010; Wilkinson *et al.*, 2016). Individual studies find that restrictions on hours of sale reduce harm ( Duailibi *et al.*, 2007; Kypri *et al.*, 2014; Rossow & Norström, 2011).	Licensing of alcohol sales outlets allows local governments to control when alcohol is sold to the public, with restrictions on hours of sale related to less harm ( de Vocht *et al.*, 2016; Wittman, 2016a; Wittman, 2016b).
Bar policies		
Training of bar staff, responsible serving practices, security staff in bars and safety- oriented design of the premise	Mixed effectiveness A systematic review found limited impact unless backed-up by police enforcement and licence inspectors ( Ker & Chinnock, 2008).	Drinking environments can be foci of alcohol- related harms ( Hughes & Bellis, 2012). Ongoing enforcement is the required ingredient to reduce harm in drinking environments ( Brännström *et al.*, 2016; Florence *et al.*, 2011; Månsdotter *et al.*, 2007; Wallin *et al.*, 2001; Warpenius *et al.*, 2010; Trolldal *et al.*, 2013).
Advertising policies		
Volume of advertising	Effective Systematic reviews find associations between volume of advertising exposure and alcohol-related consumption and harm ( Bryden *et al.*, 2012; Booth *et al.*, 2008; Gallet, 2007; Stautz *et al.*, 2016).	Cities have the opportunity of restricting advertising, including billboards, in the public places that they own or through the public services, such as transportation, that they provide ( Fullwood *et al.*, 2016; Swensen, 2016).
Drink-drive restrictions		
Sobriety checkpoints and unrestrictive (random) breath testing	Effective Systematic reviews and meta-analyses find that both introducing and expanding sobriety checkpoints and random breath testing result in reduced alcohol-related injuries and fatalities ( Bergen *et al.*, 2014; Erke *et al.*, 2009; Shults *et al.*, 2001), enhanced with mass-media campaigns ( Elder *et al.*, 2004; Yadav & Kobayashi, 2015).	Cities have the opportunity to step-up sobriety checkpoints and random breath testing ( Voas, 2008).
Designated driver campaigns	Ineffective A systematic review did not find evidence for designated driver programmes in reducing the prevalence of people drink driving or being a passenger with a drink driver ( Ditter *et al.*, 2005)	Whist a seemingly attractive approach, there is insufficient evidence to warrant widespread investment in designated driver campaigns.
Screening, advice and treatment		
Digital interventions	Effective A systematic review found that digital interventions were just as effective as face-to-face interventions in reducing alcohol consumption and related harm ( Beyer *et al.*, 2015; Kaner *et al.*, 2015).	Off-the-shelf applications can be deployed at city level ( Crane *et al.*, 2015; Garnett *et al.*, 2015), enhanced with context awareness and use of ecological momentary assessments ( Freisthler *et al.*, 2014; Morgenstern *et al.*, 2014; Wray *et al.*, 2014).
Primary health care	Effective Systematic reviews and meta-analyses find a positive impact of screening and brief advice programmes on alcohol consumption, mortality, morbidity, alcohol-related injuries, alcohol-related social consequences, healthcare resource use and laboratory indicators of harmful alcohol use ( O’Donnell *et al.*, 2014). There is stronger evidence of effectiveness for primary health care-based screening and brief advice programmes than for emergency care ( Nilsen *et al.*, 2008), general hospital settings ( McQueen *et al.*, 2011), obstetric or antenatal care ( Doggett *et al.*, 2005), and pharmacy settings ( Brown *et al.*, 2016). Systematic reviews and meta-analyses find that implementation strategies are effective in increasing the volume of primary health care screening and brief advice activity ( Anderson *et al.*, 2004; Keurhorst *et al.*, 2015).	Tailored screening and brief advice programmes embedded within community and municipal action are more likely to be scaled-up ( Anderson *et al.*, 2017; Heather 2006).
Workplace	Largely ineffective Systematic reviews of workplace-based programmes ( Webb *et al.*, 2009) and workplace-based screening and brief advice programmes find little evidence for reducing consumption and harm ( Schulte *et al.*, 2014).	Although business cases are made for workplace-based programmes ( Martinic, 2015), the evidence appears insufficient to justify a city- based investment.
Secondary health care	Effective Systematic reviews find that psycho-social ( Magill & Ray, 2009; Smedslund *et al.*, 2011; The British Psychological Society & The Royal College of Psychiatrists, 2011) and pharmacological therapies ( Rösner *et al.*, 2010a; Rösner *et al.*, 2010b; The British Psychological Society & The Royal College of Psychiatrists, 2011) are effective in treating heavy drinking.	Treatment services can be embedded within comprehensive care pathways ( NICE, 2016) at the city level.
Education and information		
School-based programmes	Ineffective Systematic reviews find that reported benefits are seen only in the short term and are often not replicated ( Foxcroft & Tsertsvadze, 2011a; Strom *et al.*, 2014)	Whilst a popular intervention, and a necessary part of school education, investment in school- based education programmes should be proportionate, given the evidence for lack of effectiveness.
Public information campaigns	Ineffective Systematic reviews find evidence of little or no sustained impact of public education campaigns in changing drinking behaviour ( Martineau *et al.*, 2013), with the exception of drink driving ( Elder *et al.*, 2004).	Media campaigns should focus on changing behaviour in relation to existing programmes, such as drink driving ( Yadav & Kobayashi, 2015), rather than acting in isolation, where there is evidence of ineffectiveness.
Changing social norms	Limited evidence Overviews suggest that alcohol-related social norms can be changed by campaigns, particularly when related to behaviour changes ( Miller & Prentice, 2016; Anderson *et al.*, 2018).	Social norms campaigns should focus on topics that are the subject of behaviour change programmes, such as drink driving ( Perkins *et al.*, 2010).
Product reformulation		
Alcohol content and packaging	Limited evidence A systematic review indicates the theoretical likelihood that reductions in the average alcohol content of beverages would reduce alcohol-related harm ( Rehm *et al.*, 2016).	Cities could set limits on beverage container sizes ( Jones-Webb *et al.*, 2011; McKee *et al.*, 2012).

## Discussion

Cities can be natural units for promoting health ( de Leeuw *et al.*, 2015; Farrington *et al.*, 2015), including reducing the harmful use of alcohol ( Mathrani & Anderson, 1998). Although not necessarily having the full jurisdictional responsibilities of national governments for all health policy issues, they often have greater flexibility and are an important site for innovative environmental measures that make healthier choices easier choices, shifting social norms in the process ( Reeve *et al.*, 2015). Cities are members of many networks, including Healthy Cities networks, which are natural vehicles for deployment to full scale globally.

We have undertaken this review to investigate the potential impact of comprehensive city and municipal-based action to reduce the harmful use of alcohol amongst adults. We have addressed adults, rather than adolescents and young people for two reasons. The first reason is that, notwithstanding the neurotoxicity of alcohol to brain development, leading in adolescence to structural hippocampal changes ( Squeglia *et al.*, 2014), the bulk of alcohol-related morbidity and mortality occurs in middle age ( Office for National Statistics, 2015). Actions that reduce the harmful use of alcohol amongst adults can lead to almost immediate reductions in health burden and risk of premature death, for injuries and alcohol use disorders, as well as for a number of chronic conditions, including cardiovascular diseases and liver cirrhosis ( Rehm *et al.*, 2011; Rehm & Roerecke, 2013). For reductions in harmful use of alcohol in adolescents, however, were they sustained, they would take many years before they impact on middle-aged morbidity and mortality. In fact, there is no available evidence which demonstrates that reductions in adolescent harmful use of alcohol lead to reductions in adult alcohol-related morbidity and mortality ( McCambridge *et al.*, 2011). The second reason for addressing adults is that there is a paucity of evaluated actions to reduce alcohol-related harm amongst adults at the community and municipal level, whereas this is not the case for actions addressing adolescents and young people (for example, see: Foxcroft & Tsertsvadze, 2011a; Foxcroft & Tsertsvadze, 2011b; Foxcroft & Tsertsvadze, 2011c; Hingson & White, 2014; Jones *et al.*, 2007; Jones, 2014; Korczak *et al.*, 2011; Newton *et al.*, 2017; Spoth *et al.*, 2008). A dissonance in research between adult oriented and youth-oriented actions to reduce the harmful use of alcohol (see Martineau *et al.*, 2013) may reflect an undue focus of existing alcohol policy on youth-oriented actions ( Anderson *et al.*, 2012; Madureira-Lima & Galea, 2018), despite their lack of evidence for impact ( Burton *et al.*, 2017; Spoth *et al.*, 2008). Implementation of what are described as politically palatable alcohol policies ( Nelson *et al.*, 2014), such as those targeting youth, increased during the first decade of the 21st century at the expense of policies that would address adults in both the United States ( Nelson *et al.*, 2014) and the European Union ( Anderson, 2013).

Our review has identified a suite of evidence-based policies and programmes that could, dependent on jurisdictional responsibilities, be implemented at the city level to reduce the harmful use of alcohol amongst adults. Indeed, some of the studies identified by the reviews that focused on one particular programme area, for example enforcement of bar regulations, stepped up drink-driving activities, and strengthened access regulations, found some impacts in reducing the harmful use of alcohol. However, only one study evaluated a suite of interventions, and found no convincing evidence of impact. This study, though, was unable to include a range of potential impactful interventions (price and availability), and did not report implementation fidelity of the included interventions Shakeshaft *et al.*, 2012).

We have not been able to find evidence for the effective impact of comprehensive municipal action plans in reducing the harmful use of alcohol amongst adults. There is, thus, dissonance between calls for action and evidence. This contrasts with other topic areas, such as smoking ( Moreland-Russell *et al.*, 2016; Perlman *et al.*, 2016), obesity ( Reeve *et al.*, 2015; Sisnowski *et al.*, 2016), and physical activity ( Giles-Corti *et al.*, 2016; Sallis *et al.*, 2016a; Sallis *et al.*, 2016b; Stevenson *et al.*, 2016), where there is experience of coordinated city action and a supporting evidence base.

We conclude our paper by discussing how this dissonance might be reduced. First, there are needs for multi-city studies that test the impact of developed and implemented municipal action plans in reducing the harmful use of alcohol. To match the WHO target of a 10% reduction in the harmful use of alcohol over a 15-year time frame, recognizing the importance of addressing adults, and, in particular heavy drinkers, the evidence base indicates that municipal action plans need to include, where jurisdictional authority allows, all of: sales taxes to increase the price of alcohol; reductions in the availability of alcohol through restrictions on outlet density and days and hours of sale; intensive implementation of drink drive restrictions through sobriety checkpoints and/or unrestrictive (random) breath testing; and, widespread deployment and scale-up of health care based screening and brief advice and treatment programmes, see Table 1. Municipal action plans should not be based on public education or mass media programmes alone, as these have been found to be ineffective in reducing the harmful use of alcohol ( Martineau *et al.*, 2013).

Ideally, multi-city studies should be set up as randomized trials with control cities in different sites with sufficient sample size with respect to both cities and individuals within cities to test whether the municipal action plans work. While this may be difficult to realize, given that such randomized trials would have very high costs, and there are not many examples of sufficiently powered community trials in the literature, a different design in combining national aggregate and individual level data could be used to achieve better control ( Gmel *et al.*, 2004). Longitudinal individual level data could be collected by drawing representative samples from the adult population in the cities, with an oversampling of heavier drinkers. This would allow an analysis of how the city actions affect individual drinking trajectories, as recommended by a number of authors ( Fitterer *et al.*, 2015; Holmes *et al.*, 2015). The evaluation should triangulate the individual cohort data with the aggregate-level data, and with other routinely collected data, such as alcohol-attributable hospitalizations ( Shakeshaft *et al.*, 2014) to test if the municipal actions lead to a reduction in the harmful use of alcohol. Wastewater-based epidemiology can be used to contrast alcohol consumption between implementation cities and comparator cities ( Ryu *et al.*, 2016). Logic models should be developed to undertake process evaluation ( Moore *et al.*, 2015) to ascertain implementation fidelity and to identify and empirically demonstrate effective best practices to reduce harmful consumption of alcohol that could be adopted for scale-up in other cities and countries ( Barker *et al.*, 2016).

## Data availability

The data referenced by this article are under copyright with the following copyright statement: Copyright: © 2018 Anderson P et al.

Data associated with the article are available under the terms of the Creative Commons Zero "No rights reserved" data waiver (CC0 1.0 Public domain dedication). 

All data underlying the results are available as part of the article and no additional source data are required.
