# Supplementary material for: City-based action to reduce harmful alcohol use: review of reviews
Source: F1000Res. 2018 Mar 5;7:120. Originally published 2018 Jan 29. [Version 2] doi: 10.12688/f1000research.13783.2 (PMC5843824; doi:10.12688/f1000research.13783.2)
Supplement: Peer review history

# Peer review: Referee response for version 2

**DOI:** 10.5256/f1000research.15435.r31472

**Published:** 2018-03-08

**Authors:** R.H.L.M Bovens

**Affiliations:** 1 Tilburg University, Tilburg, Netherlands

The authors have sufficiently responded to my comments.

I have read this submission. I believe that I have an appropriate level of expertise to confirm that it is of an acceptable scientific standard.

# Peer review: Referee response for version 1

**DOI:** 10.5256/f1000research.14982.r31220

**Published:** 2018-02-26

**Authors:** R.H.L.M Bovens

**Affiliations:** 1 Tilburg University, Tilburg, Netherlands

I think this paper contributes very much to the discussion what to do about reducing alcohol problems in society among adult people.

 It shows in the world there is little effort to focus these problems in an integrative way, using evidence based interventions as the ‘three best buys’: reducing advertisement, availability and raising taxes.

 Authors did their job accurate.

 I just have one comment:

 The paper focuses on adult people. In the research were, as far as I know only reviews that address adults.

 Why do the authors do not discuss the gap between the policy used focussing youth and the policy focussing adults?

 For example the research carried out by Karen Schelleman at your own university shows small evidence for effect of city-based actions in the Netherlands (Karen Schelleman-Offermans, R. Knibbe, E. Kuntsche: Preventing Adolescent Alcohol Use: Effects of a Two-Year Quasi-Experimental Community Intervention Intensifying Formal and Informal Control. Journal of Adolescent Health 54(3) November 2013; Karen Schelleman-Offermans, R. Knibbe, M. Derickx & H. van de Mheen: A Process Evaluation of a Community Intervention to Reduce Youth Drinking. Sucht 59(5):249-259 · January 2013).

 The thing is many countries are focusing only on youth, I think that is the main reason there are so few researches about community interventions in the adult world.

 This is the reason why I chose ‘Partly’ in the first question: accurate: Yes, but in my view not complete.

 Paying attention to the gap between the policy regarding youth and regarding adults would improve the paper seriously.

I have read this submission. I believe that I have an appropriate level of expertise to confirm that it is of an acceptable scientific standard, however I have significant reservations, as outlined above.

# Response

**Published:** 2018-03-01

**Authors:** Peter Anderson

**Affiliations:** Newcastle University , UK

Author response: Thank you for this important comment. We have added a paragraph to the discussion that stresses, from a health perspective, the importance of addressing adults as opposed to adolescents. We noted that there is considerably more research that focuses on community-based action to reduce the harmful use of alcohol among adolescents rather than among adults and suggested that this is due to an undue focus of existing alcohol policy on youth-oriented actions, that have tended to strengthen during the first decade of the 21 ^st^ century, at the expense of policies that would address adults.

# Peer review: Referee response for version 1

**DOI:** 10.5256/f1000research.14982.r30366

**Published:** 2018-02-05

**Authors:** Joan Ramón Villalbí

**Affiliations:** 1 Public Health Agency of Barcelona (ASPB), Biomedical Research Centre Network for Epidemiology and Public Health (CIBERESP), Health and Life Sciences Unit, Pompeu Fabra University, Barcelona, Spain

This is a relevant paper providing needed perspectives on interventions to reduce the harm caused by alcohol. It is presented as a review of reviews, but in fact it goes a bit beyond it, as the contents of table 1 are in themselves a valuable side-product.

 I think the paper is acceptable to be indexed in its current form, and may be useful to many practitioners.

I have read this submission. I believe that I have an appropriate level of expertise to confirm that it is of an acceptable scientific standard.
